# Alginate-Mediated Synthesis of Hetero-Shaped Silver Nanoparticles and Their Hydrogen Peroxide Sensing Ability

**DOI:** 10.3390/molecules25030435

**Published:** 2020-01-21

**Authors:** Sneha Bhagyaraj, Igor Krupa

**Affiliations:** Center for Advanced Materials, Qatar University, P.O. Box 2713, Doha, Qatar

**Keywords:** alginate, synthesis, silver nanoparticles, heterostructure, sensing

## Abstract

A new method for the simple synthesis of stable heterostructured biopolymer (sodium alginate)-capped silver nanoparticles (Ag-NPs) based on green chemistry is reported. The as-prepared nanoparticles were characterized using the ultraviolet-visible (UV-Vis) absorption spectroscopy, X-ray diffraction (XRD), transmission electron microscopy (TEM), Fourier transform infrared spectroscopy (FTIR), and dynamic light scattering (DLS) techniques. The results showed that the as-prepared Ag-NPs have a heterostructured morphology with particle size in the range 30 ± 18–60 ± 25 nm, showing a zeta potential of −62 mV. The silver nanoparticle formation was confirmed from UV-Vis spectra showing 424 nm as maximum absorption. The particle size and crystallinity of the as-synthesized nanoparticles were analyzed using TEM and XRD measurements, respectively. FTIR spectra confirmed the presence of alginate as capping agent to stabilize the nanoparticles. The Ag-NPs also showed excellent sensing capability, with a linear response to hydrogen peroxide spanning a wide range of concentrations from 10^−1^ to 10^−7^ M, which indicates their high potential for water treatment applications, such as pollution detection and nanofiltration composites.

## 1. Introduction

Silver nanoparticles have been the focus of extensive research for many decades due to their unique physical, chemical, and electrical properties, which make them suitable for diverse applications, such as catalysis, electrochemical, and biomedical applications, and thus have attracted the interest of many researchers over time [1,2]. One among the main challenges regarding the synthesis of silver nanoparticle is to prepare uniform and stable silver colloids in aqueous media [3]. In order to avoid agglomerations, metal ions are usually reduced in presence of protective colloids like water soluble biopolymers [4], water insoluble biopolymers [5], and enzymes [6]. Among the synthesis techniques for silver nanoparticles, green synthesis protocols, which can be used to prepare stable and unique nanostructures, are gaining more attention [7,8,9]. Naturally derived materials such as biopolymers could be a good alternative to toxic reagents such as trisodium citrate and borohydrides for use in synthesis methods due to the absence of biohazards associated with their byproducts and subsequent waste management [10,11,12].

Alginate is a naturally occurring polysaccharide composed of linear polymer chains of α-l-guluronate (G) and β-d-mannuronate (M) units in such an arrangement that the copolymer units appear to be in the form of an irregular block pattern. The biodegradability and biocompatibility of alginate, together with its ability to form gels upon reacting with divalent cations, make it highly useful in various biological applications, such as developing 3D tissue/organs for tissue engineering, wound healing scaffolds, and drug delivery platforms [13,14]. The presence of negatively charged carbonyl groups makes alginate highly water soluble, which makes it suitable for the synthesis of various metal nanostructures [15]. The ionotropic nature of metal alginate gels and the macromolecular polymer chains of alginate has the ability to chelate metal ions resulting in the formation of various nanoscale arrays of metals [16].

Various biopolymers have been reported as capping and reducing agents for the synthesis of silver nanoparticles. Most of the literature reports demonstrate the formation of small, spherical silver nanoparticles in the presence of various biopolymers, such as starch, gelatin, and cellulose [17,18,19]. Heterostructured silver nanoparticles are reported mainly while following the plant mediated synthesis protocols [20], using stabilizers which has the ability to limit the growth of certain crystallographic planes of nanoparticles [21] and in some physical synthesis protocols [22]. Sodium alginate has also been used as a capping and reducing agent for the synthesis of silver nanoparticles [23]. However, earlier reported methods for the synthesis of alginate capped silver nanoparticles used hydrolyzing agents such as NaOH to initiate the reaction and control the pH of the medium. In these base hydrolyzed synthesis methods, the resultant nanoparticles were morphologically homogenous in nature.

Being an inevitable part of various manufacturing industries, hydrogen peroxide (H_2_O_2_), which is a reactive oxygen species, whose higher concentration in water cause adverse effects on humans and marine ecosystems. According to US Environmental Protection Agency (EPA), the allowed limit of residual H_2_O_2_ in drinking water is 25–50 ppm. Various analytical methods like spectroscopy [24], chemiluminescence, chromatographic techniques [25], electrochemical sensors [26], photo-electrochemical sensors [27], etc., have been used to detect H_2_O_2_. Sensitive and accurate measurement of H_2_O_2_ using environmentally friendly reagents and simple equipment is a promising field of research.

Herein, we report a new, green, and facile route for synthesizing sodium alginate-silver nanoparticles (Ag-NPs) having heterostructured morphologies using alginate and glucose as the capping and reducing agents, respectively. No additional hydrolyzing agents were used in the whole reaction. Another advantage of the reported method is the use of moderate physical conditions such as atmospheric pressure and absence of any inert atmosphere which increases the feasibility of the process. The formation of Ag-NPs was confirmed using various spectroscopic techniques. Furthermore, the capability of the as- synthesized Ag-NPs towards the sensing of hydrogen peroxide is also tested. The as-synthesized Ag-NPs showed excellent sensing activity towards the presence of hydrogen peroxide even at a very low concentration of 10^−7^ M.

## 2. Results

**Figure 1:**
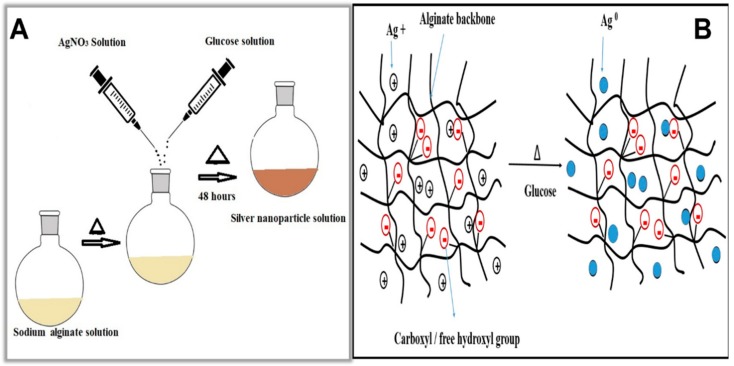
(**A**) Schematic representation of the synthesis of sodium alginate-capped silver nanoparticles (Ag-NPs). (**B**) Schematic representation of the formation of sodium alginate-capped Ag-NPs.

A simple, environmentally benign method in which a natural polymer sodium alginate used as capping agent and glucose as reducing agent to synthesize heterostructured alginate-capped Ag-NPs is presented. A schematic illustration of the synthesis protocol for alginate-capped AgNPs is given in Figure 1A. From the addition of AgNO_3_ solution to the alginate solution, the stirring speed of the reaction was maintained at 250 rpm throughout the reaction. After the addition of glucose solution to the alginate/Ag+ solution, the color of the reaction solution changes from light yellow to light brown after 1 h of reaction and then to dark brown after 40 h of reaction, indicating the formation of glucose-reduced alginate-capped silver nanoparticles. Schematic representations of the formation of alginate-capped Ag-NPs are shown in Figure 1B. Due to the presence of a large number of carboxyl (RCOO-) and free hydroxyl (OH-) groups in the polymer skeleton, sodium alginate shows a high negative charge density, which facilitates the capture of Ag+ by the polysaccharide chains, where it undergoes reduction. The formation of larger particles by coalescence is restricted by the steric hindrance and electrostatic repulsion provided by the presence of sodium alginate, thereby providing a stabilizing effect on the Ag-NPs.

### 2.1. UV-Visible Spectra Analysis of Ag-NPs

**Figure 2:**
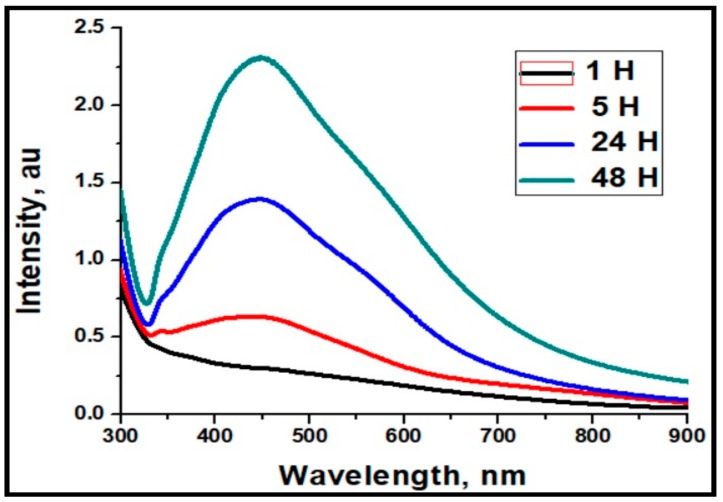
UV absorption spectra of alginate-capped Ag-NPs at different reaction times.

Figure 2 presents UV-visible absorption spectra of the as-prepared Ag-NPs at various reaction times. The absorption spectra clearly show the characteristic surface plasmon resonance (SPR) peaks of the silver nanoparticles, and the shift in the absorption maxima from 436 to 424 nm can be related to the variation in particle size during the course of the reaction. The nature of SPR bands depends on various factors like size, morphology, dielectric characteristics, etc. [28]. During initial hours of the reaction, the absorption peak intensity is very low, and the peak is very broad which indicates that the number of particles is low with larger particle size. As the reaction time increases, the absorption intensity increases together with a blue shift in absorption wavelength indicating the formation of smaller particles. From the absorption spectra, it is clear that the absorption peak of the Ag-NPs was broadened to longer wavelengths, indicating the presence of nonspherical particles [29]. The as synthesized alginate capped Ag-NPs were found to be stable at normal atmospheric conditions even after 4 months.

### 2.2. FTIR Analysis of Ag-NPs

**Figure 3:**
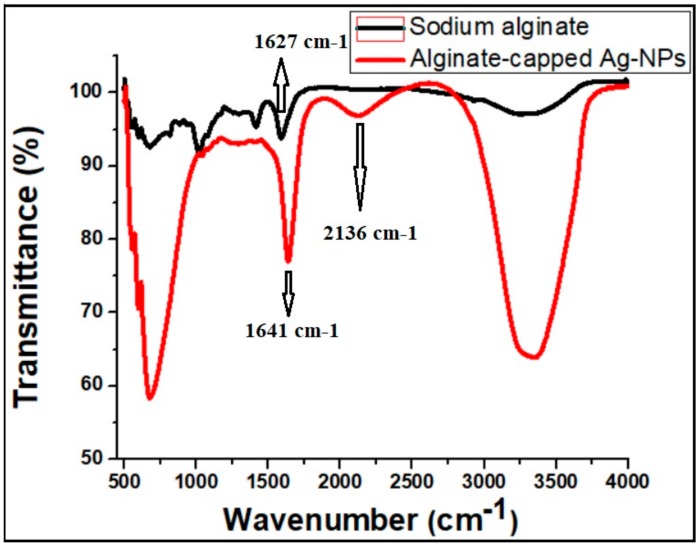
Fourier transform infrared spectroscopy (FTIR) spectra of the Ag-NPs.

In the FTIR spectrum of sodium alginate in Figure 3, the broad peak at approximately 3325 cm^−1^ represented the stretching vibrations of the hydroxyl groups (-O-H). The minor peak at 1045 cm^−1^ can be assigned to the C-O vibrations from the alginate backbone. The significant peak at 1641 cm^−1^ is due to the C-O stretching of the -COOH group of the alginic acid unit. In the FTIR spectrum of alginate- capped Ag-NPs, a more intense hydroxyl peak is visible around 3352 cm^−1^. Since Ag-NPs are in the colloidal form with water as a component, this result is justified. The weak broad band at 2136 cm^−1^ in the spectrum of the alginate-capped Ag-NPs may be due to –CO-Ag linkages [30]. The C-O-C stretching mode arising from the glucosidic units gives rise to the absorption band at 1083 cm^−1^. Compared to the FTIR spectrum of sodium alginate, the spectrum of the alginate-capped Ag-NPs exhibits visible shifts in the absorption peaks of the CO_2_− (from 1627 to 1641 cm^−1^) and O-H groups (from 3325 to 3352 cm^−1^). These shifts can be considered as an indication that the carboxyl groups and hydroxyl groups are responsible for the synthesis and stabilization of the Ag-NPs.

### 2.3. XRD Analysis

**Figure 4:**
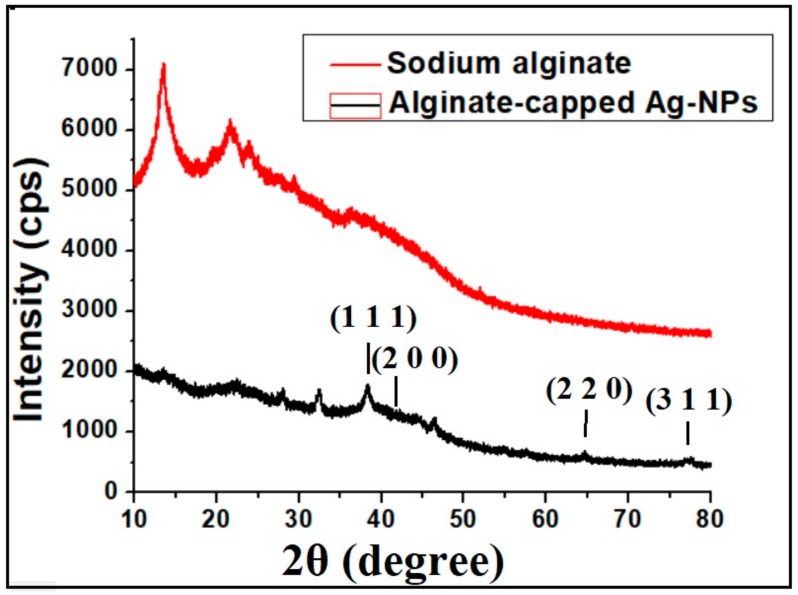
X-ray diffraction pattern of sodium alginate and alginate-capped Ag-NPs after 48 h reaction time.

Figure 4 shows the X-Ray diffraction patterns of neat sodium alginate polymer and alginate-capped Ag-NPs. X-ray diffraction (XRD) data indicates that sodium alginate used in this study shows a semi-crystalline nature. The crystalline structure of the as-synthesized alginate-capped silver nanoparticles is clearly visible from its XRD pattern. The diffraction profile of all the Ag-NPs were similar having XRD peaks in the range of 2θ (30° < 2θ < 80°). Strong Bragg reflections at 38.21°, 44.23°, 64.48°, and 77.51° were observed corresponding to the (111), (200), (220), and (311) planes, respectively, of the face-centered cubic crystalline structure of metallic silver (JCPDS No. 01-1167). From the XRD pattern, it was clear that the main phase is silver pointing to the absence of any major impurities [31].

### 2.4. Zeta Potential Measurements

**Figure 5:**
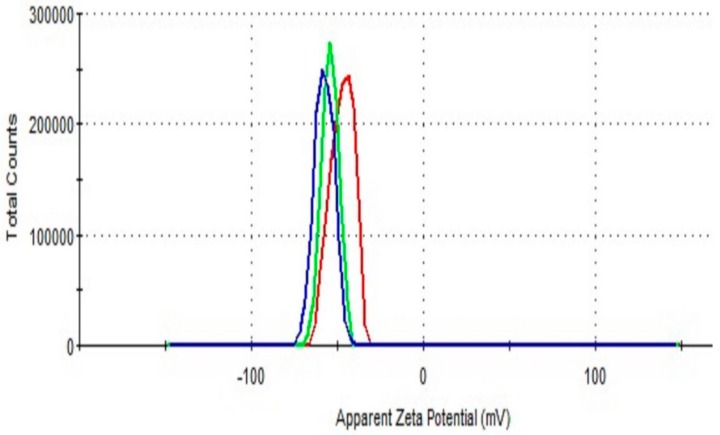
Zeta potential distribution of the Ag-NPs at a reaction time of 48 h.

Zeta potential measurements indicate the stability of nanoparticles in aqueous suspensions. Figure 5 presents the results of zeta potential analysis of the as-synthesized alginate-capped Ag-NPs at 48 h. The Ag-NPs exhibited a negative zeta potential with a value of −62 mV, which is in the range required for a stable suspension. This finding clearly indicates that the Ag-NPs have high surface charges, which endow them with electrostatic stability to prevent aggregation, thereby improving their stability.

### 2.5. TEM and HRTEM Measurements

**Figure 6:**
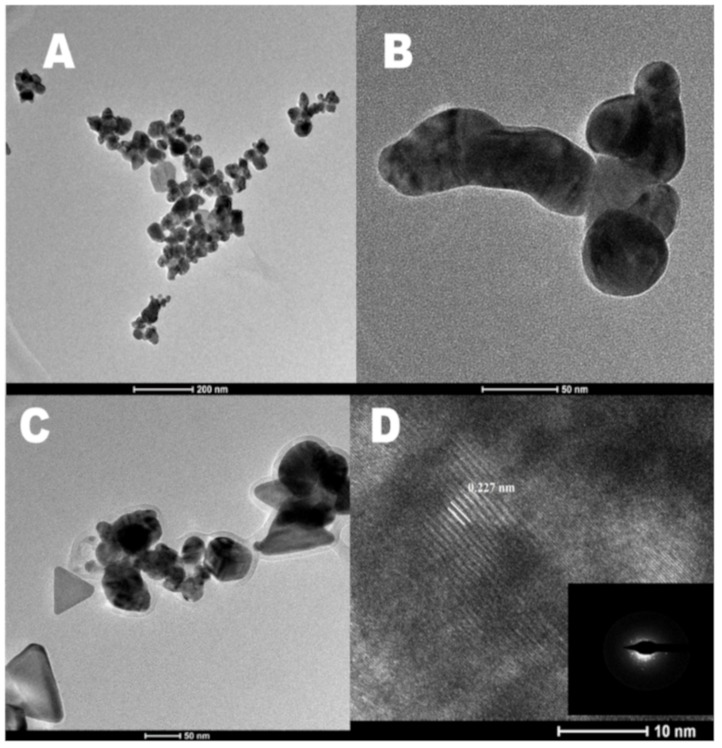
Transmission electron microscopy (TEM) images of alginate-capped silver nanoparticles at (**A**) 1 h, (**B**) 24 h, and (**C**) 48 h. (**D**) HRTEM image of Ag-NPs at 48 h along with the corresponding SAED pattern (inset).

Transmission electron microscopy (TEM) images of the as-synthesized Ag-NPs at different reaction times are shown in Figure 6. During the initial hours of the reaction, the density of the silver particle is too high, and the alginate capping is minimum. This results in the aggregation of the particles formed. This is clearly visible from the TEM image of Ag-NPs at 1 h as shown in Figure 6A. Compared to other biopolymers commonly used in metal nanoparticle synthesis, sodium alginate is an anionic polymer which can interact and reduce the Ag^+^ to Ag^0^ due to the presence of a large number of carboxyl and hydroxyl groups in it [32]. Hence, interaction of metal ions with the polymer chains and the polymer–polymer interaction causes the initial particles formed to aggregate focusing on specific crystal planes resulting in the formation of larger particles. From Figure 6B which is the TEM image of the Ag-NPs at 24 h reaction time, it is visible that the particles start to grow in certain crystal planes. These particles act as seed particles to form heterostructured Ag-NPs after 48 h of reaction. The presence of different shapes of Ag-NPs is observed in Figure 6C which is the TEM image of Ag-NPs at 48 h reaction time. The particle size of the Ag-NPs during the reaction was determined from the TEM images using ImageJ software and was found to be between 30 ± 18 (1 h) and 60 ± 25 nm (48 h). The High Resolution Transmission Electron Microscope (HRTEM) image of the Ag-NPs at 48 h is shown in Figure 6D. The crystal planes of the face centered cubic structured Ag-NPs are clearly visible with an interplanar distance measuring 0.227 nm. The Selected Area Electron Diffraction (SAED) pattern (inset) also highlights the crystallinity of the as prepared Ag-NPs.

### 2.6. Hydrogen Peroxide Sensing Measurements

**Figure 7:**
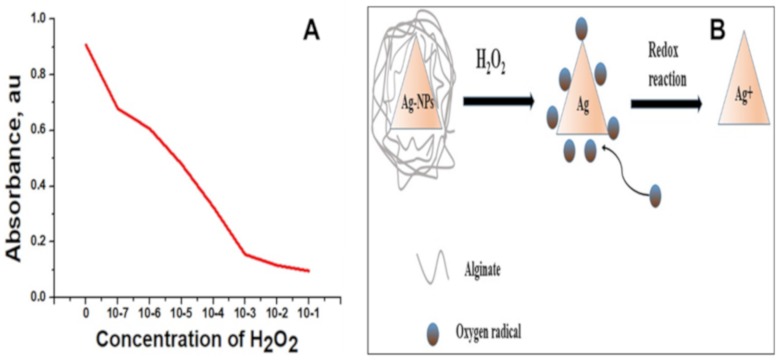
(**A**) Change in absorbance versus H_2_O_2_ concentration. (**B**) Possible mechanism of the reaction between the Ag-NPs and H_2_O_2_.

The oxidative degradation followed by the depolymerization of sodium alginate in the presence of reactive oxygen species like H_2_O_2_ is reported [33]. The effect of various smaller concentrations of H_2_O_2_ on the absorption behavior of as-synthesized alginate-capped Ag-NPs was analyzed in this experiment. The variation in absorbance intensity of Ag-NPs in the presence of H_2_O_2_ at different concentrations after 60 s of reaction is shown in Figure 7A. For the experiment, 48 h Ag-NPs solutions with the absorption maximum of 436 nm was used. The addition of H_2_O_2_ to the Ag-NPs solution caused the destruction of the alginate capping layer covering the Ag-NP surface, which facilitates nanoparticle aggregation [34]. The absorption maxima show a blue shift during the interaction with H_2_O_2_ and were shifted from 436 to 416 nm with 10^−7^ M H_2_O_2_. The catalytic reaction between Ag and H_2_O_2_ is visible, as the color of the solution changes gradually from yellow to colorless depending on the concentration of H_2_O_2_. The absorbance intensity decreases with increasing H_2_O_2_ concentration. Compared to other biopolymer-capped Ag-NPs, the as-synthesized alginate-capped Ag-NPs showed improved sensitivity towards H_2_O_2_ even up to a concentration of 10^−7^ M, which is in line with previously reported data from our group [35]. A possible mechanism for the sensing activity is schematically presented in Figure 7B. Reactive oxygen species producing compounds such as H_2_O_2_ cause the degradation of the biopolymer-capped Ag-NPs initially by depolymerizing the alginate capping followed by the oxidation of Ag to Ag+, which results in a decrease in the localized surface plasmon resonance (LSPR) absorbance.

## 3. Materials and Methods 

### 3.1. Materials

All the chemicals used were of analytical grade and were used without any further purification. AgNO_3_ was obtained from Loba Chemie (Mumbai, India), while sodium alginate (alginic acid sodium salt from brown algae), glucose, and H_2_O_2_ (30% solution) were obtained from Sigma Aldrich, Beijing, China.

### 3.2. Method

Alginate-capped Ag-NPs synthesis: the synthesis method used is a modified procedure from our reported work [35]. For the reaction, 1.0 g of sodium alginate powder was added to 100 mL of distilled water in a round bottom flask, and the resulting solution was heated to 60 °C with vigorous stirring to obtain a clear solution. After 60 min, 5 mL of AgNO_3_ solution (1 M) was added to the alginate solution with continuous stirring to obtain Ag+/alginate solution. This step was followed by the addition of 10 mL of glucose solution (0.08 M) under continuous stirring. The reaction was maintained at 80 °C and run for 48 h. Aliquots were taken at different time intervals (1, 5, 24, and 48 h) to monitor the growth of the particles.

Sensing studies: the sensing activity was studied using a reported procedure with slight modifications [36]. To analyze the sensing properties of the Ag-NPs against H_2_O_2_, silver nanoparticles (48 h sample) were added to different concentrations of hydrogen peroxide solutions (2000 µL) in a quartz cuvette at the ratio of 1:2. The reaction between the nanoparticles and H_2_O_2_ for 60 s was monitored using the absorption spectra. The change in the ultraviolet–visible (UV–vis) spectrum with varying concentrations of H_2_O_2_ in the range from 10^−1^ to 10^−7^ due to the catalytic reaction between the silver nanoparticles and hydrogen peroxide was carefully analyzed.

### 3.3. Characterizations

A UV spectrophotometer (Biochrom Libra S60, Cambridge, UK) was used to measure the absorption behavior of the Ag-NPs in the range of 200–900 nm. For analyzing the UV, the sample was diluted with water in the ratio 1:2. FT-IR was done using a PerkinElmer Spectrum 400 spectrophotometer (Waltham, MA, USA) in the range 400–4000 cm^−1^ with a resolution of 2 cm^−1^. For measuring FT-IR, the sodium alginate powder and the alginate-capped Ag-NPs solution were used. For zeta potential measurements (Malvern Zeta size analyzer, UK), the sample was diluted with water in the ratio 1:2 to avoid errors. X-ray diffraction analysis was performed using a diffractometer (PANalytical model X’PERT-PRO, Malvern, UK) with Kα radiation of 1.5418 Å. The Ag-NPs solution was dried in the oven and crushed using a mortar to prepare fine powdered nanoparticles for XRD measurements. TEM and HRTEM measurements were performed using a JEOL JEM-3010 electron microscope (Japan) operating at 200 kV. For TEM analysis, the sample was prepared by casting a single micro drop of the Ag-NPs solution on a TEM grid and dried.

## 4. Conclusions

An innovative, simple, and green method for the synthesis of stable heterostructured sodium alginate-capped silver nanoparticles is reported. Sodium alginate was used as the capping agent, while glucose acted as the reducing agent. The as-synthesized Ag-NPs were small (30 ± 18–45 ± 12 nm) and spherical during the initial (1–10) hours of the reaction and evolved to become heterostructured after 48 h with the particle size reaching 60 ± 25 nm. The shape and crystallinity of the as-synthesized Ag-NPs were confirmed by XRD, TEM, and HRTEM analyses. The alginate-capped Ag-NPs showed excellent sensing activity towards the addition of hydrogen peroxide even at a very low concentration of 10^−7^ M H_2_O_2_. Detecting pollutants at low concentrations in water requires high sensitivity, and developing nanomaterials using a green synthesis protocol for this purpose is highly significant. Alginate-capped Ag-NPs synthesized using a green protocol can also be tested for their potential applications in biomedical and food processing applications.
